# Recurrent Pneumonia due to Fibrosing Mediastinitis in a Teenage Girl: A Case Report with Long-Term Follow-Up

**DOI:** 10.1155/2018/3246929

**Published:** 2018-03-18

**Authors:** Avigdor Hevroni, Chaim Springer, Oren Wasser, Avraham Avital, Benjamin Z. Koplewitz

**Affiliations:** ^1^Institute of Pulmonology, Hadassah-Hebrew University Medical Center, Jerusalem, Israel; ^2^Department of Radiology, Hadassah-Hebrew University Medical Center, Jerusalem, Israel

## Abstract

A teenage girl was evaluated for recurrent right pneumonia. The evaluation revealed a calcified mediastinal mass that compressed the right intermediate and middle lobar bronchi, as well as the right pulmonary artery and veins. The clinical picture together with imaging studies and borderline positive serology testing suggested a diagnosis of fibrosing mediastinitis associated with histoplasmosis. This rare condition is characterized by the local proliferation of invasive fibrous tissue within the mediastinum due to a hyperimmune reaction to *Histoplasma capsulatum*. Antifungal and anti-inflammatory therapies are usually ineffective, and surgical intervention contains a high morbidity risk. Palliative surgery and stenting of the compressed airway have been suggested. In the past, the prognosis was thought to be poor, but recent studies demonstrate a more positive outcome. Our patient had been radiologically and functionally stable under follow-up for over thirteen years and has married and delivered two healthy children, both following an uneventful pregnancy.

## 1. Introduction

Fibrosing mediastinitis (FM) is a rare disorder characterized by the proliferation of locally invasive fibrous tissue within the mediastinum. Affected patients suffer from signs and symptoms related to the compression of vital organs located within the mediastinum. Although the etiology of FM remains unclear, most cases have been related to immune-mediated hypersensitivity reactions to antigens from the fungus *Histoplasma capsulatum*, which is endemic to North and Central America [1–3]. In the past, the prognosis was considered poor [1]; however, our case and other recent reports suggest a more favorable prognosis [2]. Here, we will present the evaluation and follow-up of a teenage girl presented with recurrent right lobe pneumonia which was finally diagnosed as FM.

## 2. Case Report

A thirteen-year-old girl was admitted to our hospital due to recurrent right pneumonia throughout the previous three years. She lost 3 kg in body weight in the preceding weeks but did not have fever or night sweats. She was born in Israel to parents of North African descent, with her mother completing treatment for Hodgkin's lymphoma 1.5 years before the girl's admission. During the years 1997–1999, when the patient was between six and eight years old, the family lived in Panama, Mexico and Florida.

Upon physical examination, she appeared well and had no signs of respiratory distress. Lung auscultation revealed decreased breath sounds over the right lung. There was no clubbing, and the rest of the exam was unremarkable. The basic laboratory results were insignificantly remarkable with no significant elevation of inflammatory markers.

**Figure 1:**
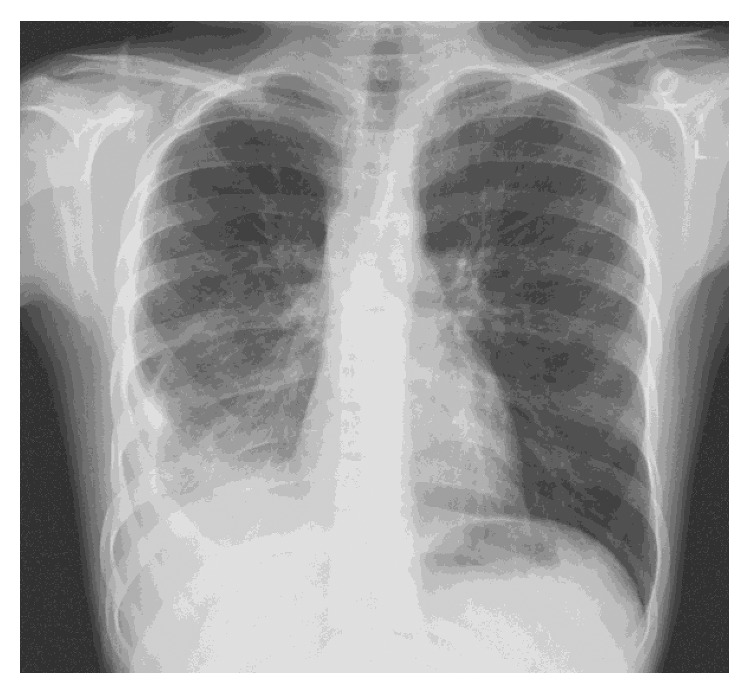
Posteroanterior chest radiography at presentation showing right lower lobe consolidation with pleural thickening and effusion, resulting in marked right lower lobe volume loss.

A chest radiograph showed consolidation in the right lower lobe (RLL), with pleural thickening and effusion, and with resultant marked right lower lobe volume loss (Figure 1).

The recurrence of pneumonia focusing in the areas of the right middle and lower lobes (RML & RLL), demanded elimination of an anatomical disorder of the airways or foreign body aspiration; therefore, a direct anatomical investigation was accomplished by bronchoscopy. The bronchoscopy revealed severe obstruction of the right bronchus intermedious and a complete obstruction of the bronchus to the RML due to external compression.

Bronchoalveolar lavage (BAL) sampling was done to look for relevant infectious etiologies. Bacterial cultures, acid-fast staining, and silver staining were all negative for bacteria, tuberculosis, or fungus.

**Figure 2:**
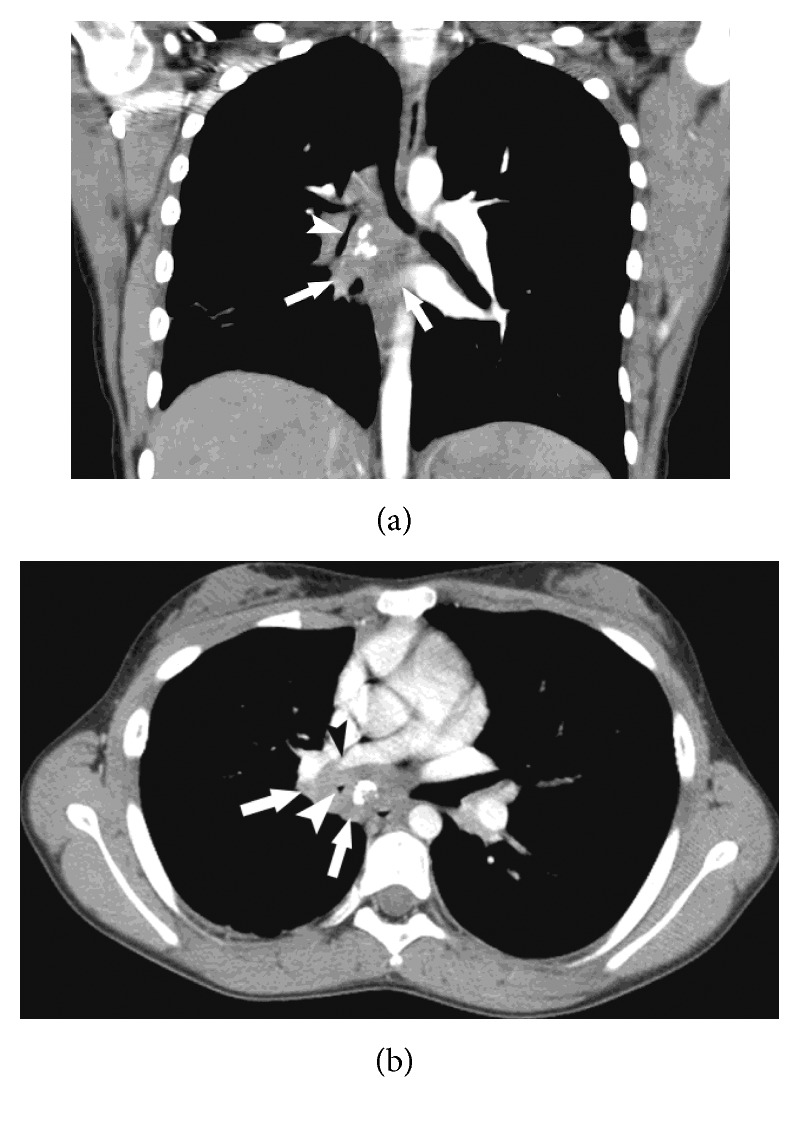
Axial (a) and coronal (b) reformats of the chest CT following a contrast injection, showing a subcarinal and right hilar ill-defined soft tissue mass (white arrows) with prominent interior calcifications causing marked compression on the bronchus intermedius, right middle lobe bronchus (white arrowhead), and right inferior pulmonary vein (black arrowhead).

A chest computed tomography (CT) was done for assessing the external compression shown by bronchoscopy and for ruling out additional relevant, though less probable, etiologies for recurrent right pneumonia. Additional etiologies for recurrent right pneumonia include some congenital anomalies such as congenital pulmonary airway malformation or a diaphragmatic hernia. The chest CT showed subcarinal and right hilar masses (3.6 and 2 cm, resp.) with prominent calcifications. These calcifications severely compressed the right bronchial tree, right pulmonary artery, and right inferior pulmonary vein (RPA and RPV), causing complete atelectasis of the RML (Figure 2).

In the differential diagnosis of the calcified mass, the first to be considered were tuberculosis, fungal infection, and a malignancy. Other options included nodule calcification due to a granuloma formation in response to a healed infection, for example, a healed varicella pneumonia [4]. It may have also represented the noncaseating granulomatous disease such as sarcoidosis or primary pulmonary amyloidosis. Another rare nonmalignant tumor to be considered was pulmonary chondroma as a part of the Carney triad which typically affects young females [5]. Last but not the least, calcification of the lung parenchyma may be present secondary to calcium and phosphate metabolism abnormalities [6] or to a benign inflammatory stimulus leading to calcifying fibrous pseudotumour (CFPT) of the lung [7].

An elaborated series of tests were completed due to suspected tuberculosis. PPD (Mantoux test) was negative as was the mother's (as a possible source). A quantiferon test and gastric fluid culture yielded the same results. Specimens of bronchoalveolar lavage fluid, pleural fluid, and pleural biopsy all came back negative for TB as well as for malignancy, granulomas, fungi, or other pathologies. In addition to a pleural biopsy, a biopsy from the calcified mass was also obtained by a transbronchial biopsy. Though the biopsy detected a few inflammatory cells alongside fibrous tissue, it did not reveal any infectious pathogens, granulomas, or other specific pathologies.

Extensive evaluation showed negative results for other relevant infectious etiologies (except for what will be mentioned below), immune deficiencies, and autoimmune diseases. Serum angiotensin-converting enzyme activity was also within normal limits.

The patient's previous residence in areas endemic for histoplasmosis (U.S. and Mexico) gave us a clue to histoplasmosis infection as a possible source for our findings. Histoplasmosis antibody tests (performed by the Centers for Disease Control and Prevention (CDC) in Atlanta, Georgia, United States) were weakly positive with regard to the complement fixation test (titer of 1:8) and negative for the immunodiffusion test.

The clinical picture, imaging studies [8], serology results (although borderline), and past residence in areas endemic for histoplasmosis together with negative findings of other possible etiologies, all pointed to the diagnosis of histoplasmosis-related FM.

A series of complementary tests were done to better demonstrate the compression, to evaluate the severity of the lung disease, and for quantitative follow-up.

**Figure 3:**
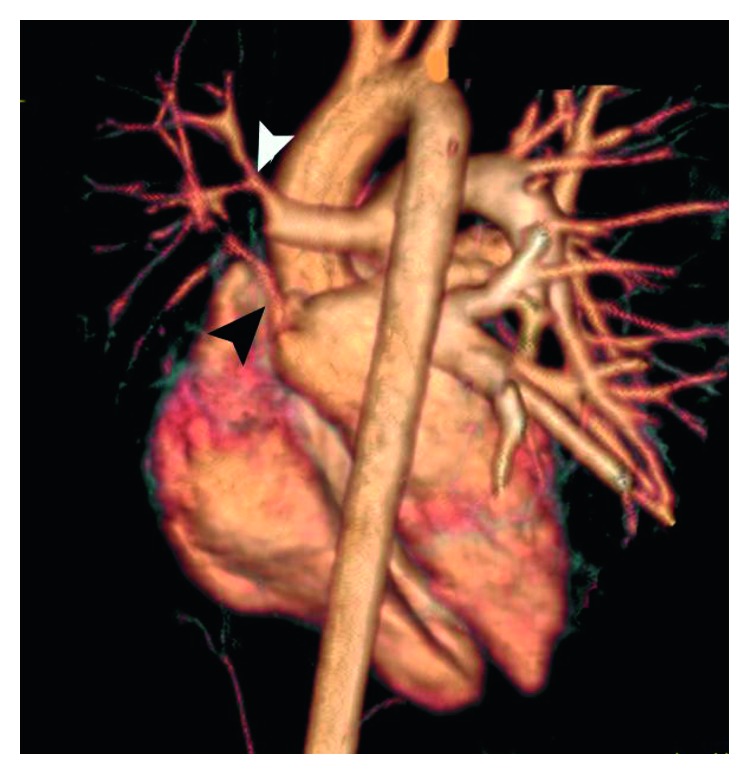
3D reconstruction of the cardiac MRI demonstrates marked narrowing of the right pulmonary artery (black arrowhead) and the right inferior pulmonary veins (white arrowhead).

Cardiac magnetic resonance imaging (MRI) demonstrated normal ventricular function and narrowing of the RPA and RPV (Figure 3). Pulmonary function testing (PFT) revealed a mildly restrictive pattern with normal CO diffusion to alveolar gas (DL_CO_/VA). On a perfusion/ventilation scan (V/Q), the ventilation scan showed a mild ventilation defect at the RT lower lobe, while the perfusion scan showed a severe perfusion defect in the middle and lower RT lobes. Cardiopulmonary exercise test (CPET) results displayed suboptimal peak O_2_ consumption with a normal respiratory reserve.

### 2.1. Treatment

After ruling out a malignancy, but before TB test results returned, antitubercular treatment was commenced along with prednisone for two weeks due to the pleural involvement and the bronchial obstruction. As soon as the TB test results were found to be negative, the patient was slowly weaned off this treatment.

After the diagnosis of histoplasmosis-related FM became the most probable diagnosis, we started treatment with itraconazole (200 mg twice a day) for three months. In addition, several trials of bronchoscopic balloon dilatation of the right intermedious bronchus were performed but achieved only transient relief.

### 2.2. Follow-Up

Presently, after 13 years of follow-up, the patient functions normally regarding daily tasks but experiences some limitations during strenuous physical activity. She experiences annual or sometimes biannual symptoms of a chest infection which are treated with antibiotics.

Upon physical examination, she shows no signs of respiratory distress, with an SpO_2_ of 100% on room air and good air entry to both lungs.

Throughout the follow-up period, no significant changes in the classified mass's size or impact were documented on CT scans. MRI images also showed no change. Lung function tests and physiologic respiration abilities on CPET remain intact compared to baseline.

The patient is now married and has delivered two healthy children, both with regular vaginal deliveries following an uncomplicated pregnancy, under tight multidisciplinal supervision.

## 3. Discussion

Histoplasmosis is a fungal infection resulting from the inhalation of spores of the fungus *Histoplasma capsulatum* which is endemic to the Ohio and Mississippi river valleys in the United States and also to Mexico and Central and South America. An estimated 40 million people in the United States have been infected with *H. capsulatum*, with 500,000 new cases occurring each year. Still, even in these localities, FM is relatively rare.

Transmission occurs by the mycelial form of *H. capsulatum* which is found in the soil. When spores produced by the mycelial form of *H. capsulatum* become airborne, they are inhaled and are deposited in the alveoli. At normal body temperature (37°C), the spores germinate into the yeast form of this dimorphic fungus and are ingested by pulmonary macrophages. The yeasts become parasitic, multiply within these cells, and travel to hilar and mediastinal lymph nodes where they gain access to the blood circulation and are disseminated to various organs.

The diagnosis is easier by visualizing the yeast in tissue or by culture. The majority of infected persons is asymptomatic or has mildly symptomatic, self-limiting illnesses. However, in some situations significant manifestations may develop [9].

There are several types of serious histoplasmosis-related pulmonary conditions. The first is *acute diffuse pulmonary histoplasmosis* which occurs in patients exposed to a large number of infectious spores. The symptoms develop within a week or two and can progress to respiratory failure or progressive extrapulmonary dissemination. Most patients recover without treatment, but some of them remain dyspneic and fatigued for months. Conversely, *chronic pulmonary histoplasmosis* follows exposure in patients with underlying lung diseases. The clinical and radiographic findings resemble those of reactivated tuberculosis. The progressive disease process that ends in necrosis and loss of lung tissue results from a hyperimmune reaction to fungal antigens rather than from the infection itself. The symptoms can progress to cavities, formation of bronchopleural fistula, aspergilloma, atypical mycobacterial infections, chronic or recurrent pneumonia in areas of lung damage, and concurrent neoplasms in 5% of cases [2]. The third type, *disseminated pulmonary histoplasmosis*, is a rare disease which occurs primarily in immunocompromised patients [9], especially in patients with HIV infections. The spectrum of this illness is variable and ranges from a chronic, intermittent course in immunocompetent persons to an acute and rapidly fatal infection in infants and severely immunosuppressed people. In the fourth type, *granulomatous mediastinitis*, the mediastinal lymph nodes are enlarged, encapsulated, and caseous, following a direct infection by *H. capsulatum*. The last condition, *fibrosing mediastinitis* (FM), is also known as sclerosing mediastinitis. This is a benign but often progressive and potentially lethal disorder, characterized by infiltration of dense fibrous tissue into mediastinal fat [2].

A small number of patients with histoplasmosis develop FM. The average interval separating the initial symptoms from diagnosis is 3.5 years although in most cases the acute episode of histoplasmosis is not identified. Affected patients suffer from signs and symptoms related to the compression of the vital organs located within the mediastinum such as the superior vena cava (SVC), pulmonary veins, and arteries, trachea, bronchi, or esophagus. CT scans show a localized, calcified mass in the paratracheal or subcarinal regions of the mediastinum or the pulmonary hila. This mass may represent an idiosyncratic fibroinflammatory reaction to previous *H. capsulatum* infection. FM is also attributed to tuberculosis, sarcoidosis, autoimmune diseases, and radiation therapy. It may also be idiopathic. However, due to different clinical courses and the pathology itself, the aforementioned causes of FM are considered a different disease type than FM of histoplasmosis origin.

Diagnosis is usually based on the clinical picture and on characteristic imaging studies. Serologic studies are of limited diagnostic value. A biopsy can be hazardous, and when achievable, it is not always diagnostic. Therefore, it is usually reserved for excluding other diagnoses.

There is probably no curative therapy for FM to date. Antifungal and anti-inflammatory treatments such as glucocorticoids are generally ineffective, with only few reports suggesting benefits [10]. Surgical treatment presents a high risk of morbidity and mortality due to the hard fibrous mass caused by FM which consolidates the vital mediastinal structures. Lobectomy, pneumonectomy, stenotic airway resection, reconstruction, or bypass of involved arteries and veins may be offered to relieve symptoms. Bronchoscopic stent placement in the airways or percutaneous stent placement in blood vessels such as the SVC and pulmonary arteries or veins allow symptomatic relief but often necessitate reintervention [11]. Recently, reports from the Mayo Clinic demonstrated improvement in progressive FM following treatment with rituximab. After the researchers had reported accumulation of CD20-positive B lymphocytes in FM tissue, they considered treatment with rituximab, a monoclonal antibody targeted against CD20. Twelve patients with FM complicated by vascular or airway compromise along with increased metabolic activity by positron emission tomography with fluoro-D-glucose (FDG PET) test were treated with rituximab. All of them demonstrated symptomatic and radiologic improvement following this treatment, as predicted [12, 13].

Previous case reports and case series of FM reported a poor prognosis with a high mortality rate [1]. However, in a recent publication describing the course of 80 adult patients with FM, with an average follow-up of 41 months, the mortality rate was similar to the age-matched controls [2].

Presently, 13 years since the primary diagnosis, our patient is under no specific treatment; her mediastinal mass is stable, and she has only minor limitations with regard to exercise. She is married, has given birth to two healthy children, and lives a relatively normal life.
